# Validation of Reference Genes for Gene Expression Normalization in RAW264.7 Cells under Different Conditions

**DOI:** 10.1155/2019/6131879

**Published:** 2019-05-16

**Authors:** Zhenzhen Bao, Yanli Huang, Jiyu Chen, Zhenglong Wang, Jiang Qian, Jiyang Xu, Yucheng Zhao

**Affiliations:** ^1^School of Pharmacy, Jiangsu Health Vocational College, Nanjing, Jiangsu, China; ^2^School of Life Science and Technology, China Pharmaceutical University, Nanjing, Jiangsu, China; ^3^Jiangsu Key Laboratory of Bioactive Natural Product Research and State Key Laboratory of Natural Medicines, School of Traditional Chinese Pharmacy, China Pharmaceutical University, Nanjing, Jiangsu, China

## Abstract

RAW264.7 is a macrophage strain derived from mice tumour and shows a significant ability in antigen uptake. Real-time quantitative PCR (RT-qPCR) is one of the most commonly used methods in gene studies and requires suitable reference genes to normalize and quantitate the expression of gene of interest with sensitivity and specificity. However, suitable reference genes in RAW264.7 cells have not yet been identified for accurate gene expression quantification. In the current study, we evaluated expression levels of ten candidate reference genes in RAW264.7 cells under different conditions. RT-qPCR results indicated significant differences in the expression levels among the ten reference genes. Statistical analyses were carried out using geNorm, NormFinder, and BestKeeper software to further investigate the stability of the reference genes. Integrating the results from the three analytical methods, cytochrome c-1 and hydroxymethylbilane synthase were found to be the most stable and therefore more suitable reference genes, while ribosomal protein L4 and cyclophilin A were the least stable. This study emphasises the importance of identifying and selecting the most stable reference genes for normalization and provides a basis for future gene expression studies using RAW264.7 cells.

## 1. Introduction

Reverse transcription quantitative real-time PCR (RT-qPCR) is an important method for gene expression studies [1, 2]. This technology has become a very popular method owing to its high speed, high sensitivity, and high-throughput capabilities [3–5]. However, the results are inevitably affected by sample variation and PCR efficiency, which could lead to erroneous interpretations. Hence, in order to ensure accurate measurement of the expression levels of genes in various conditions, normalization of target gene expression with that of a proper reference gene is absolutely necessary when using RT-qPCR [6–8]. Several reference genes, including glyceraldehyde-3-phosphate dehydrogenase (GAPDH), beta-2-microglobulin (B2M), and *β*-actin (ACTB), have been reported for their stable expression in all tissues and cells [9, 10]. Considering that gene expression levels may vary among cells or tissues and may also change under certain circumstances, geNorm [11], NormFinder [12], and BestKeeper [13] analytical software have been specially designed for screening of reference gene stability.

Macrophages are important immune cells and play a critical role in anti-infection, antitumour, and immune regulation processes. A large number of cell lines of murine macrophages, such as P338D1 and J774A1 cells, have been commonly used in the study of microbiology and immunology [14, 15]. In contrast, RAW264.7 cells, macrophages derived from mice tumour, have rarely been examined for suitable candidate reference gene expression in a variety of drug treatments [16]. Hence, in this study, we chose RAW264.7 cells to evaluate the expression levels of reference genes for reliable normalization under different conditions. Conversely, a number of studies have shown that no single reference gene exists that can be expressed stably under any experimental condition [17]. Therefore, ten reference genes, ACTB, GAPDH, ribosomal protein L4 (RPL4), hypoxanthine phosphoribosyltransferase-1 (HPRT1), cyclophilin A (PPIA), Cytochrome c-1 (CYC1), hydroxymethylbilane synthase (HMBS), eukaryotic translation elongation factor 1 alpha 1 (Eef1a1), glucuronidase *β* (GUSB), and lactate dehydrogenase A (LDHA), were selected and analysed for suitability under different conditions [9, 10, 18–24].

This study utilized the three analytical methods mentioned above to examine the stability of multiple commonly used reference genes in RAW264.7 cells. To the best of our knowledge, this is the first study to systematically evaluate the expression stability of candidate reference genes in RAW264.7 cells under different conditions (various drugs and concentrations). We believe that the present work will provide a substantial foundation for future research in mouse or human cells.

## 2. Materials and Methods

### 2.1. Cell Culture and Treatment

Murine macrophage cells, RAW264.7, were grown in DMEM (Gibco, USA) supplemented with 100 U/mL penicillin-streptomycin and 10% foetal bovine serum (FBS, Bioind) and incubated at 37°C in a 5% CO_2_ humidified atmosphere. Upon confluence, cells were trypsinised and treated, respectively, with various drugs at multiple concentrations: high-glucose (HG; 50 mM, 100 mM, 200 mM), hydrogen peroxide (H_2_O_2_; 50 *μ*M, 100 *μ*M, 200 *μ*M), lipopolysaccharide (LPS; 0.1, 0.5, 1 *μ*g/mL), cobalt chloride (CoCl_2_; 50 *μ*M, 100 *μ*M, 200 *μ*M), and palmitic acid (PA; 50 *μ*M, 100 *μ*M, 200 *μ*M). Cells without treatment (WT) acted as the control group. Three biological repeats were used for each condition.

### 2.2. PCR Primers for Reference Genes

Ten reference genes (ACTB, GAPDH, RPL4, HPRT1, PPIA, CYC1, HMBS, Eef1a1, GUSB, and LDHA) of RAW264.7 cells were selected for evaluation based on the high frequency of their use in other related studies [9, 10, 18–24]. Primers used for determining the expression of the ten reference genes are listed in Table 1. According to the manufacturer, these primers were designed and optimized using Primer 5 as follows: primer length, 18–22 bp; GC content, 40%–60%; amplification length, 100–150 bp, to avoid the formation of primer-dimer during the reaction.

### 2.3. RNA Extraction and cDNA Synthesis

RNA was extracted from the treated and control RAW264.7 cells using RNAiso Plus total RNA kit (TransGen Biotech, Dalian, China). DNase I (Takara, Dalian, China) treatment was carried out to purify RNA. The RNA samples with OD_260_/OD_280_ ratios between 1.8 and 2.0 and a total amount of 0.5 *μ*g were used for cDNA synthesis to ensure the precision of the trial. Additionally, the purification of the RNA was confirmed by agarose gel electrophoresis. The cDNA was synthesised from the RNA using reverse transcriptase and the HiScript® Q RT SuperMix for qPCR Kit (Vazyme, Nanjing, China), by following manufacturer's instructions, and stored at –20°C until use for subsequent reaction.

### 2.4. Quantitative Real-Time PCR Analysis

The RT-qPCR reactions were set up using cDNA, forward/reverse primer, and Hieff™ qPCR SYBR® Green Master Mix (Yeasen, Shanghai, China), according to manufacturer's instructions. The reactions were carried out in 96-well PCR reaction plates in a LightCycler 480 system (Roche Molecular Biochemicals, Mannheim, Germany).

### 2.5. Data Analysis

The stability of reference genes was analysed using the three statistical software programs: geNorm [11], NormFinder [12], and BestKeeper [13]. The geNorm measures gene expression stability according to the values of M, which were calculated from the cycle threshold (Ct) values obtained from RT-qPCR. M values indicated the pairwise variation between individual gene and the other reference genes; lower values of M represented higher expression stabilities, while a higher M value indicated a more unstable reference gene. In addition, geNorm could also determine the optimal number of candidate reference genes required for normalization based on pairwise variation. Like geNorm, NormFinder tended to rank the stability of reference genes according to the M values, with the lowest M value indicating the most stable gene. BestKeeper, an Excel-based statistical method that analyses expression variability of reference genes, ranked the stability of reference genes from most to least based on the key factors of standard deviation (SD) and coefficient of variance (CV).

## 3. Results

### 3.1. Validation of Primer Specificity

As shown in Supplementary Figure 1, PCR and subsequent agarose gel electrophoresis were used to identify the specificity of the designed primers. The single band and peak of melting curve indicated that the primers possessed good specificity (Supplementary Figure 2).

### 3.2. The Expression Levels of the Reference Genes

**Figure 1:**
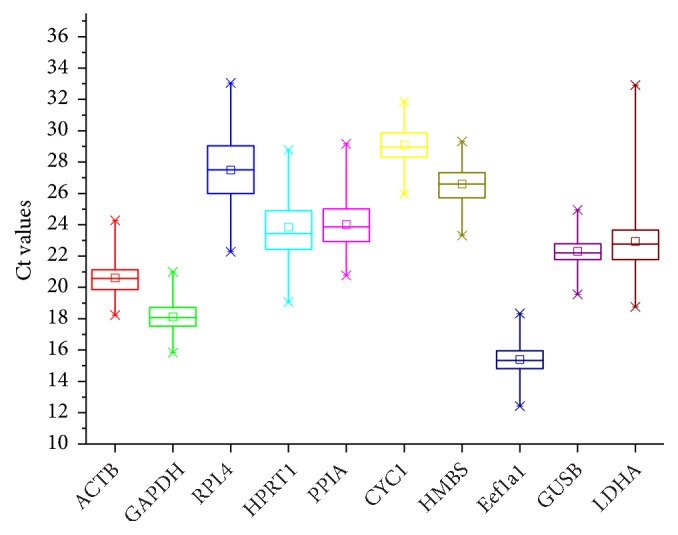
Expression levels of the ten reference genes (ACTB, GAPDH, RPL4, HPRT1, PPIA, CYC1, HMBS, Eef1a1, GUSB, and LDHA) in RAW264.7 cells. Squares of the box indicate the means; the lines indicate the median and whiskers indicate the highest and lowest values.

The Ct value generated from the RT-qPCR is the fluorescence threshold for each primer pair and reflects the expression levels of the reference genes; a low Ct value indicates high expression [4–6]. The overall mean Ct values for the ten reference genes are shown in Figure 1. Among the ten genes, there was an obvious difference in expression levels as seen from the Ct values, which ranged from 15 to 30. CYC1 was the least expressed reference gene with the highest mean Ct value, while Eef1a1, GAPDH, and ACTB were the three most stably expressed genes having lower Ct values. In addition, LDHA had a narrow range of Ct values, indicating that the variability of the expression level was constant under different conditions and it might be the best reference gene with a relatively high expression level. Similarly, ACTB and HMBS could be considered as suitable choices for stable reference genes owing to their relatively narrow Ct ranges. In contrast, RPL4 showed a large range of Ct values and would be unsuitable as a reference gene. To further systematically assess the stability of the ten reference genes under various treatments, the Ct values were transformed to analyse expression levels using the three software programs: geNorm, NormFinder, and BestKeeper.

### 3.3. GeNorm Analysis

**Figure 2:**
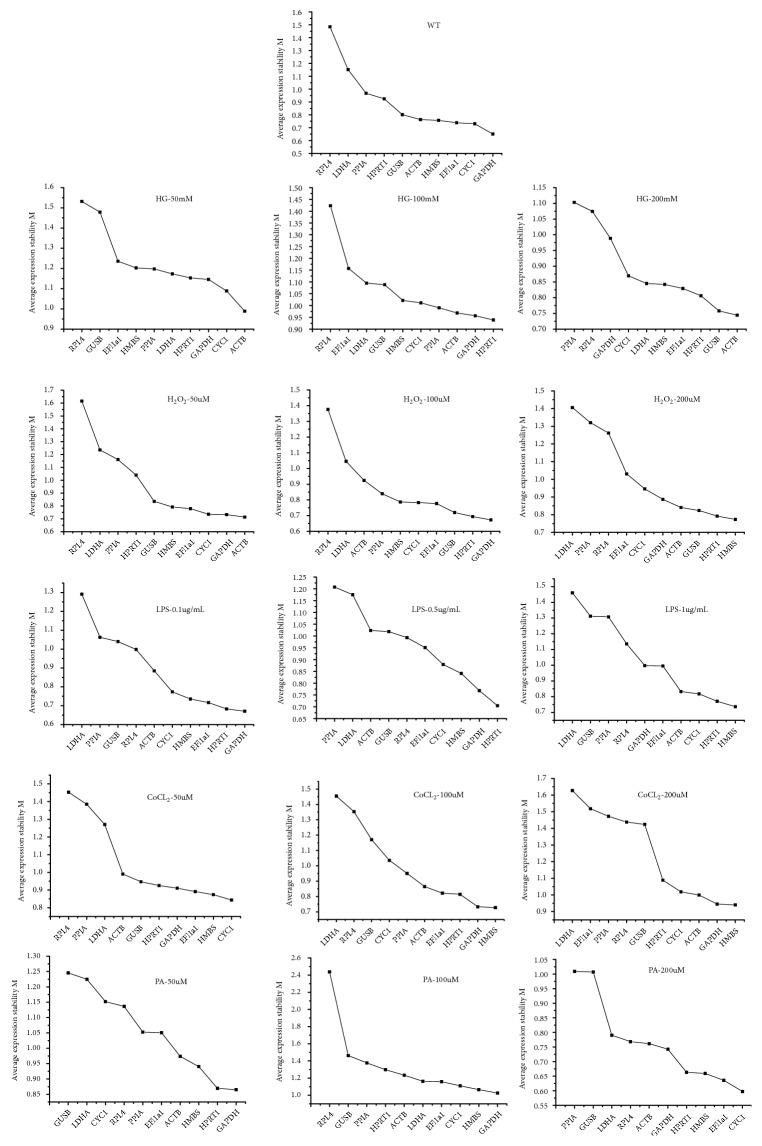
Expression stability of the reference genes analysed by geNorm. M values represent the average expression stability; the stability is ranked from left to right, indicating the stability is inversely related to the M values. The treatments of drugs and concentrations are listed in the figure correspondingly.

GeNorm used the equation 2^−ΔΔCT^ (Ct values were collected from different experimental conditions), to transform the Ct values into relative quantification data to analyse the stability of reference genes, which were ranked from the most to the least stable based on their M values. The M values were calculated by pairwise variation analysis and were considered reliable to select stable reference genes when the M values were greater than 1.5. A lower M value reflected a higher stability [11]. As shown in Figure 2, the stability rank of the ten reference genes was different under different conditions, indicating that different concentrations or drug treatments affected the gene expression. For the control group, the M values of the ten reference genes were ranked as follows: RPL4 > LDHA > PPIA > HPRT1 > GUSB > ACTB > HMBS > Eef1a1 > CYC1 > GAPDH, revealing that GAPDH was the most stable reference gene, while RPL4 was the least stable. However, for other groups, the ten reference genes had different stability ranks. For instance, the rank of the reference genes after LPS treatment (0.5 *μ*g/mL) was as follows: PPIA > LDHA > ACTB > GUSB > RPL4 > Eef1a1 > CYC1 > HMBS > GAPDH > HPRT1, indicating that, under these conditions, HPRT1 ranked the most stable reference gene, while PPIA was the least stable. In addition, different concentrations of the same drug resulted in the same gene to be ranked as the most stable reference gene; for example, ACTB was the most stable reference gene after treatment with both low and high concentrations of HG. Moreover, different drug treatments showed the same reference gene to be the most stable at similar doses of treatment. For example, HMBS was ranked the most stable candidate gene in H_2_O_2_, LPS, and CoCl_2_ treatments, respectively, but only upon treatment with high concentrations of the drugs. Taken together, these results indicated that the stability of the reference genes was not constant under different conditions. Notably, geNorm analysis indicated that GAPDH might be the most stable reference genes under various experimental conditions (drug treatments and concentrations).

### 3.4. NormFinder Analysis

The NormFinder is a statistical algorithm that calculates the M values, which are then used to rank the stability of the reference genes. Similar to geNorm, the most stable gene is associated with the smallest M value [12]. As shown in Table 2, the ten reference genes in the control group were ranked based on stability as follows: GAPDH, HMBS, CYC1, GUSB, ACTB, HPRT1, Eef1a1, PPIA, LDHA, and RPL4. Thus, GAPDH was the most expressed stable reference gene, followed by HMBS; RPL4 was the least expressed one. These results were similar to those obtained with geNorm data analysis of the control group. Interestingly, under some conditions, more than one reference gene showed the same M value, and therefore identical stability; for instance, CYC1 and HPRT1 showed the same M value and were determined to be the most stably expressed genes upon treatment with 200 *μ*M PA. Consistent with the results of geNorm analysis, the HMBS gene seemed to be the most stable reference gene, as it showed the lowest M value among the most stable genes under different conditions. However, differences were also noted between the geNorm and NormFinder analyses; for instance, CYC1 was ranked as the most stable gene by NormFinder, while geNorm ranked it as the fourth.

### 3.5. BestKeeper Analysis

Distinct from the geNorm and NormFinder methods, BestKeeper determines the gene stability based on the SD and the CV. The SD of the reference genes is a key factor to identify the most stable gene in the BestKeeper analysis; the higher the CV and SD values, the more unstable the reference gene [13]. Supplementary Table 1 shows the CV ± SD values, arranged in the decreasing order of their value from top to bottom, representing the stability rank of the reference gene from least to most. Reference genes with SD > 1.5 were eliminated for further analysis. Specifically, PPIA (SD = 1.53) in 50 *μ*M CoCl_2_, LDHA (SD = 1.56) in 200 *μ*M CoCl_2_, and RPL4 (SD = 2.50) were excluded to be able to accurately select the best normalization gene from the candidate genes. GUSB and HMBS frequently appeared as the most stable reference genes. In contrast, Eef1a1 and RPL4 often ranked as the top-most unstable genes. Notably, in the control group, GUSB had the smallest CV ± SD values of 0.34 ± 0.07, followed by CYC1 with 1.30 ± 0.36, and RPL4 had the highest expression variation with CV ± SD value of 4.13 ± 1.19. Together, the above findings indicated that BestKeeper analysis differed significantly from that of geNorm and NormFinder analysis.

### 3.6. Optimal Number of Reference Genes for Normalization

**Figure 3:**
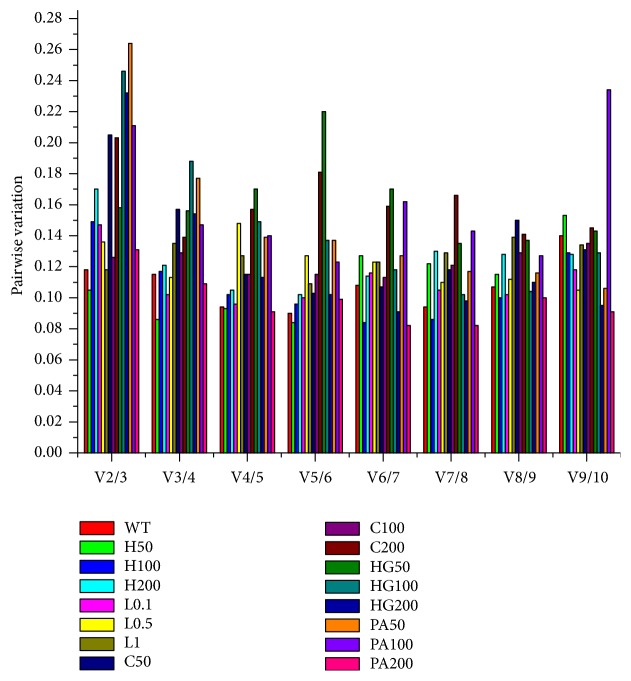
Determination of the optimal number of reference genes for normalization using geNorm analysis. Pairwise variation (Vn/n+1) of reference genes under different conditions are listed. WT, H50 (H100, H200), L0.1 (L0.5, L1), C50 (C100, C200), HG50 (HG100, HG200), and PA50 (PA100, PA200), respectively, were the abbreviation for the control group; H_2_O_2_, 50 *μ*M (100 *μ*M, 200 *μ*M); lipopolysaccharide, 0.1 *μ*g/mL (0.5, 1 *μ*g/mL); CoCl_2_, 50 *μ*M (100 *μ*M, 200 *μ*M); high-glucose, 50 mM (100 mM, 200 mM); and palmitic acid, 50 *μ*M (100 *μ*M, 200 *μ*M).

GeNorm can select the most stable expression gene and also determine the optimal number of reference genes for accurate normalization according to the pairwise variation (Vn/n+1), which is calculated from the two sequential normalization factors (NFn) and uses Vn/n+1 < 0.15 as a criterion to confirm the optimal number of reference genes for accurate analysis. The results of Vn/n+1 are shown in Figure 3. Most of the V2/3 values were greater than 0.15, indicating that using only two most stably expressed genes would not improve the accuracy under all conditions. According to the cut-off value of 0.15, the control group, H_2_O_2_ (50 *μ*M, 100 *μ*M, 200 *μ*M), LPS (0.1, 0.5, 1 *μ*g/mL), 100 *μ*M CoCl_2_, and 200 *μ*M PA could be assessed using only two suitable reference genes, while three reference genes were required under conditions of 200 *μ*M CoCl_2_ and 100 *μ*M PA, and four were required under conditions of HG (50 mM, 100 mM, 200 mM), 50 *μ*M CoCl_2_, and 50 *μ*M PA.

## 4. Discussion

It is critical to select the proper reference genes for normalization when using RT-qPCR quantification for analysing gene expression [25–27]. Our study evaluated the stability of ten reference genes in RAW264.7 cells by geNorm [11], NormFinder [12], and BestKeeper [13] programs, by comparing their expression levels under different conditions. The Ct values representing expression levels of the reference genes ranged from 15 to 30, in accordance with the results of most reports [4].

Based on the principle that lower Ct values indicate higher expression profile [4, 6], our results indicated that Eef1a1, GAPDH, and ACTB were the three most highly expressed reference genes, while CYC1 was the least expressed gene with Ct values being nearly 30 under conditions. In addition, owing to a narrow Ct range, which represents a stable expression profile, LDHA, ACTB, and HMBS were considered as the most stable reference genes, while RPL4 was the least stable. Our findings showed some differences between geNorm, NormFinder, and BestKeeper analyses, indicating that it is necessary to systematically analyse expression stability combining various analysis methods, as also suggested by previous reports [28–30].

GeNorm analysis indicated that GAPDH, HMBS, and HPRT1 were the three most stable genes under different experimental conditions. NormFinder ranked HMBS, CYC1, HPRTI, and GAPDH on the basis of high expression stability, while BestKeeper indicated that GUSB and HMBS could be considered as the most suitable normalization genes. Thus, the stability ranking of the candidate genes was not consistent across different analyses. Nonetheless, on comparing the outcomes of the three analytical programs, HMBS was the only one that was commonly identified by all three programs to be highly and stably expressed. Additionally, only slight differences were observed between the stability of the ten reference genes when comparing results of NormFinder and geNorm, likely due to the similar method adopted by both programs to evaluate gene expression stability. Integrating results of all three analyses, CYC1 and HMBS were considered to be the most stably expressing genes suitable for normalizing qPCR data in RAW264.7 cells.

GAPDH is a frequently used reference gene, although a number of studies have reported that it does not maintain constant expression level under different experimental conditions, and thus it is unsuitable for normalizing RT-qPCR data [31–34]. However, GAPDH was recognized as the most stable gene with a lower M value by geNorm analysis in our study, consistent with previous studies in mouse uterus cells and J774A1 murine macrophage cells [15, 35]. RPL4 and PPIA significantly ranked low, indicating that they were not suitable as normalizing genes. However, previous studies have reported that RPL4 was a highly stable gene, as its expression was not influenced by some experimental conditions [36–38]. Meanwhile, PPIA has been considered a highly expressed reference gene for normalization in macrophages [33, 37].

Considering that using a single reference gene could lead to inaccuracies during RT-qPCR quantification [39, 40], it is necessary to determine the optimal number of reference genes to be used for gene expression studies. In the current study, a pairwise variation (Vn/n+1) indicated that most groups required only two reference genes for normalization, as the V2/3 values were lower than the cut-off value of 0.15. However, the Vn/n+1 values of certain groups exceeded 0.15, suggesting the need for additional reference genes (three or four) to improve the accuracy of normalization.

## 5. Conclusions

In summary, we evaluated the stability of ten reference genes in RAW264.7 cells using three analytical approaches, namely, geNorm, NormFinder, and BestKeeper, to determine the most stable reference genes under different conditions. Our findings reveal that two reference genes are sufficient for accurate normalization in most conditions, although some cases might require more than two reference genes for accurate evaluation of gene expression levels. Importantly, our data indicate that CYC1 and HMBS are the most suitable reference genes, while RPL4 and PPIA are the most variable and unsuitable genes for normalization in RAW264.7 cells. These findings might play a crucial role in the selection of appropriate reference genes in further studies.

## Figures and Tables

**Table 1:** Information of the ten reference genes used in the real time quantitative PCR.

Gene	Description	Primer: forward/reverse (5'-3')	Length (bp)	Accession number
ACTB	*β*-actin	F: CAGGTCATCACTATTGGCAA	143	NM_007393
		R: AGGTCTTTACGGATGTCAAC		
GAPDH	Glyceraldehyde-3-phosphate dehydrogenase	F: TGCTGAGTATGTCGTGGAGT	136	NM_001289726
		R:GTTCACACCCATCACAAACA		
RPL4	Ribosomal protein L4	F: GGAAGTTGGATGAGCTGTAT	108	NM_024212
		R: TCAAGATTCTGCTAAGGTCG		
HPRT1	Hypoxanthine phosphoribosyltransferase-1	F: TAGTGAAACTGGAAAAGCCA	135	NM_013556
		R: AAGCTTTACTAGGCAGATGG		
PPIA	Cyclophilin A	F:CGGTTCCCAGTTTTTTATCT	102	NM_008907
		R: ATGGCTTCCACAATGTTCAT		
CYC1	Cytochrome c-1	F: CTAACCCTGAGGCTGCAAGA	113	NM_025567
		R: GCCAGTGAGCAGGGAAAATA		
HMBS	Hydroxymethyl-bilane synthase	F: ATGGCTCAGATAGCATGCAA	126	NM_013551
		R: GGGCTCCTCTTGGAATGTTA		
Eef1a1	Eukaryotic translation elongation factor 1 alpha 1	F: CCTACCACCAACTCGTCCAA	136	NM_010106
		R: AAAGGTAACCACCATGCCAG		
GUSB	Glucuronidase *β*	F: ATGTCCTGCTGAGAGGTGTC	121	NM_010368
		R: TCCAGCCTCTCACCAGTAGC		
LDHA	lactate dehydrogenase A	F: AACATCTCACTCCCCACAGC	128	NM_010699
		R: CTCACAGGGGTAATCGAAGC		

**Table 2:** Expression stability values of ten reference genes in RAW264.7 cells calculated by NormFinder analysis.

Rank	WT	HG 50 mM	HG 100 mM	HG 200 mM	H_2_O_2_ 50 *μ*M	H_2_O_2_ 100 *μ*M	H_2_O_2_ 200 *μ*M	LPS 0.1 *μ*g/mL	LPS 0.5 *μ*g/mL	LPS 1 *μ*g/mL	CoCl_2_ 50 *μ*M	CoCl_2_ 100 *μ*M	CoCl_2_ 200 *μ*M	PA 50 *μ*M	PA 100 *μ*M	PA 200 *μ*M
1	RPL4 0.048	EFl1a1 0.073	EFl1a1 0.067	GAPDH 0.039	RPL4 0.051	RPL4 0.043	LDHA 0.053	LDHA 0.046	EFl1a1 0.046	LDHA 0.056	LDHA 0.048	LDHA 0.058	EFl1a1 0.069	EFl1a1 0.058	RPL4 0.081	GUSB 0.042
2	LDHA 0.046	GUSB 0.057	RPL4 0.042	PPIA 0.037	LDHA 0.049	EFl1a1 0.040	EFl1a1 0.050	PPIA 0.036	PPIA 0.043	GUSB 0.050	PPIA 0.048	RPL4 0.043	LDHA 0.065	GUSB 0.048	GUSB 0.049	PPIA 0.034
3	PPIA 0.030	RPL4 0.053	LDHA 0.037	EFl1a1 0.036	PPIA 0.042	LDHA 0.039	PPIA 0.047	GUSB 0.035	LDHA 0.040	EFl1a1 0.049	RPL4 0.045	GUSB 0.041	GUSB 0.048	LDHA 0.044	EFl1a1 0.046	EFl1a1 0.034
4	EFl1a1 0.027	PPIA 0.041	GUSB 0.036	RPL4 0.031	HPRT1 0.029	ACTB 0.035	RPL4 0.033	ACTB 0.030	ACTB 0.036	PPIA 0.048	ACTB 0.031	ACTB 0.027	PPIA 0.046	RPL4 0.034	ACTB 0.040	LDHA 0.028
5	HPRT1 0.024	HPRT1 0.040	GAPDH 0.031	LDHA 0.027	EFl1a1 0.025	PPIA 0.025	GAPDH 0.027	EFl1a1 0.027	GUSB 0.036	GAPDH 0.036	GAPDH 0.029	CYC1 0.026	RPL4 0.039	CYC1 0.032	PPIA 0.040	ACTB 0.028
6	ACTB 0.023	GAPDH 0.039	ACTB 0.028	CYC1 0.020	GUSB 0.023	GUSB 0.016	ACTB 0.023	RPL4 0.022	RPL4 0.026	RPL4 0.032	EFl1a1 0.028	PPIA 0.024	HPRT1 0.028	PPIA 0.031	HPRT1 0.039	GAPDH 0.024
7	GUSB 0.023	LDHA 0.035	HMBS 0.026	HPRT1 0.020	GAPDH 0.017	CYC1 0.016	CYC1 0.022	CYC1 0.016	GAPDH 0.022	ACTB 0.013	GUSB 0.027	GAPDH 0.021	GAPDH 0.022	ACTB 0.030	LDHA 0.034	RPL4 0.021
8	CYC1 0.016	HMBS 0.030	CYC1 0.025	HMBS 0.020	HMBS 0.014	HMBS 0.015	GUSB 0.014	HPRT1 0.012	CYC1 0.020	CYC1 0.006	HPRT1 0.022	EFl1a1 0.019	CYC1 0.018	GAPDH 0.024	CYC1 0.029	HMBS 0.020
9	HMBS 0.015	ACTB 0.019	HPRT1 0.023	GUSB 0.020	CYC1 0.012	GAPDH 0.012	HPRT1 0.008	HMBS 0.010	HMBS 0.019	HMBS 0.006	HMBS 0.014	HPRT1 0.015	ACTB 0.017	HMBS 0.022	HMBS 0.029	CYC1 0.015
10	GAPDH 0.011	CYC1 0.018	PPIA 0.020	ACTB 0.018	ACTB 0.010	HPRT1 0.009	HMBS 0.006	GAPDH 0.010	HPRT1 0.010	HPRT1 0.002	CYC1 0.007	HMBS 0.001	HMBS 0.006	HPRT1 0.019	GAPDH 0.020	HPRT1 0.015

## Data Availability

The data used to support the findings of this study are included within the article.

## Supplementary Materials

Supplementary 1 Supplementary Figure 1: Agarose gel (1%) electrophoresis of the ten reference genes. 1-10 represent ACTB, GAPDH, RPL4, HPRT1, PPIA, CYC1, HMBS, Eef1a1, GUSB, and LDHA, respectively.

Supplementary 2 Supplementary Figure 2: Melt curves of the ten reference genes.

Supplementary 3 Supplementary Table 1: Expression stability values of the ten reference genes calculated by BestKeeper in RAW264.7 cells.
