# Supplementary figures and images for: Validation of Reference Genes for Gene Expression Normalization in RAW264.7 Cells under Different Conditions
Source: Biomed Res Int. 2019 May 16;2019:6131879. doi: 10.1155/2019/6131879 (PMC6541955; doi:10.1155/2019/6131879)

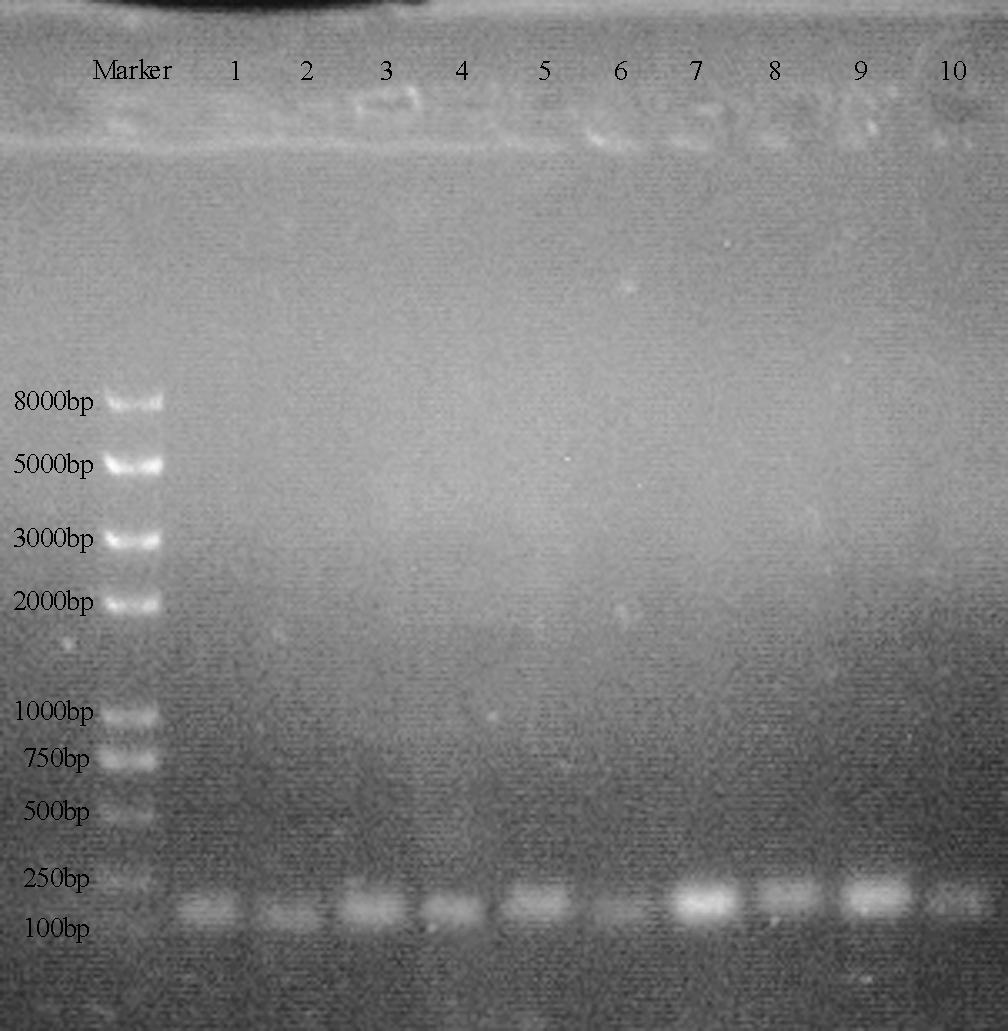

Supplement: Supplementary 1 — Supplementary Figure 1: Agarose gel (1%) electrophoresis of the ten reference genes. 1-10 represent ACTB, GAPDH, RPL4, HPRT1, PPIA, CYC1, HMBS, Eef1a1, GUSB, and LDHA, respectively. [file 6131879.f1.tif]

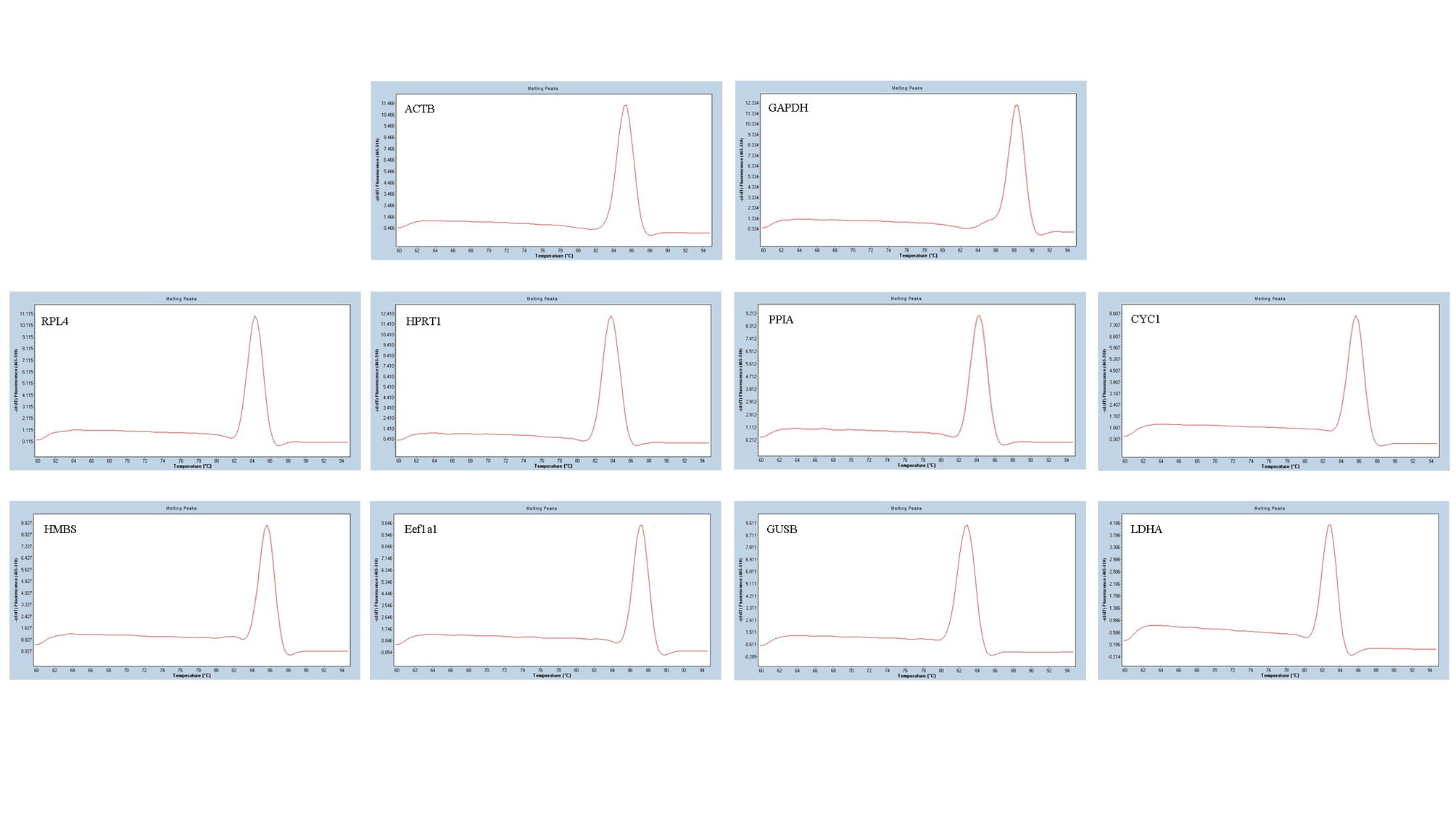

Supplement: Supplementary 2 — Supplementary Figure 2: Melt curves of the ten reference genes. [file 6131879.f2.tif]
