# Supplementary material for: Validation of Reference Genes for Gene Expression Normalization in RAW264.7 Cells under Different Conditions
Source: Biomed Res Int. 2019 May 16;2019:6131879. doi: 10.1155/2019/6131879 (PMC6541955; doi:10.1155/2019/6131879)
Supplement: Supplementary 3 — Supplementary Table 1: Expression stability values of the ten reference genes calculated by BestKeeper in RAW264.7 cells. [file 6131879.f3.doc]

**Supplementary Table 1** Expression stability values of the reference genes calculated by BestKeeper in RAW264.7 cells.

| **Rank** | **WT** | **HG**  **50 mM** | **HG**  **100 mM** | **HG**  **200 mM** | **H_2_O_2_**  **50 μM** | **H_2_O_2_**  **100 μM** | **H_2_O_2_**  **200 μM** |
| --- | --- | --- | --- | --- | --- | --- | --- |
| **1** | GUSB  0.34 ± 0.07 | ACTB  1.43 ± 0.30 | LDHA  1.92 ± 0.45 | GUSB  1.73 ± 0.39 | GUSB  0.55 ± 0.12 | GUSB  0.78 ± 0.17 | GUSB  1.05 ± 0.23 |
| **2** | CYC1  1.30 ± 0.36 | GAPDH  1.55 ± 0.28 | GUSB  2.00 ± 0.45 | LDHA  2.01 ± 0.45 | CYC1  1.03 ± 0.29 | CYC1  1.44 ± 0.42 | HPRT1  1.72 ± 0.48 |
| **3** | HMBS  2.12 ± 0.55 | HPRT1  1.62 ± 0.37 | HMBS  2.68 ± 0.72 | ACTB  2.34 ± 0.48 | ACTB  1.81 ± 0.36 | HPRT1  1.51 ± 0.42 | HMBS  2.03 ± 0.54 |
| **4** | LDHA  2.44 ± 0.53 | CYC1  1.77 ± 0.49 | CYC1  3.33 ± 1.01 | CYC1  2.51 ± 0.73 | LDHA  2.00 ± 0.44 | GAPDH  1.72 ± 0.31 | CYC1  2.54 ± 0.75 |
| **5** | HPRT1  2.56 ± 0.68 | PPIA  2.18 ± 0.52 | GAPDH  3.49 ± 0.65 | HMBS  2.72 ± 0.74 | HMBS  2.10 ± 0.55 | HMBS  1.79 ± 0.48 | GAPDH  2.87 ± 0.54 |
| **6** | GAPDH  2.95 ± 0.51 | RPL4  2.95 ± 0.86 | ACTB  3.52 ± 0.76 | GAPDH  3.02 ± 0.55 | GAPDH  2.22 ± 0.40 | ACTB  2.01 ± 0.40 | LDHA  2.92 ± 0.68 |
| **7** | Eef1a1  3.13 ± 0.47 | HMBS  3.42 ± 0.88 | HPRT1  3.83 ± 0.90 | HPRT1  3.11 ± 0.72 | Eef1a1  2.69 ± 0.41 | LDHA  2.35 ± 0.54 | ACTB  2.95 ± 0.61 |
| **8** | ACTB  3.60 ± 0.70 | LDHA  4.20 ± 0.95 | PPIA  4.22 ± 1.06 | PPIA  3.14 ± 0.78 | HPRT1  3.05 ± 0.80 | PPIA  2.80 ± 0.65 | RPL4  3.33 ± 1.02 |
| **9** | PPIA  4.02 ± 0.94 | GUSB  5.75 ± 1.29 | RPL4  5.40 ± 1.50 | RPL4  3.51 ± 0.93 | PPIA  3.91 ± 0.88 | RPL4  3.95 ± 1.17 | Eef1a1  4.30 ± 0.68 |
| **10** | RPL4  4.13 ± 1.19 | Eef1a1  6.20 ± 0.93 | Eef1a1  6.27 ± 0.98 | Eef1a1  3.73 ± 0.57 | RPL4  5.12 ± 1.44 | Eef1a1  4.07 ± 0.63 | PPIA  4.37 ± 1.04 |

| **Rank** | **LPS**  **0.1 μg/mL** | **LPS**  **0.5 μg/mL** | **LPS**  **1 μg/mL** | **CoCl_2_**  **50 μM** | **CoCl_2_**  **100 μM** | **CoCl_2_**  **200 μM** | **PA**  **50 μM** | **PA**  **100 μM** | **PA**  **200 μM** |
| --- | --- | --- | --- | --- | --- | --- | --- | --- | --- |
| **1** | GUSB  1.33 ± 0.30 | ACTB  1.46 ± 0.31 | HMBS  1.72 ± 0.46 | GUSB  1.40 ± 0.31 | HMBS  1.70 ± 0.46 | CYC1  1.48 ± 0.45 | HMBS  2.72 ± 0.71 | HMBS  1.52 ± 0.39 | HMBS  2.92 ± 0.73 |
| **2** | CYC1  2.93 ± 0.88 | HPRT1  1.61 ± 0.36 | LDHA  1.92 ± 0.45 | HMBS  1.92 ± 0.52 | HPRT1  1.71 ± 0.40 | GUSB  2.05 ± 0.47 | ACTB  2.81 ± 0.56 | CYC1  2.22 ± 0.63 | CYC1  3.09 ± 0.86 |
| **3** | GAPDH  3.25 ± 0.62 | HMBS  1.78 ± 0.48 | ACTB  2.18 ± 0.45 | LDHA  1.98 ± 0.44 | Eef1a1  2.05 ± 0.32 | HMBS  2.33 ± 0.65 | GUSB  2.92 ± 0.64 | ACTB  2.87 ± 0.59 | GAPDH  3.28 ± 0.56 |
| **4** | LDHA  3.44 ± 0.87 | CYC1  2.09 ± 0.62 | CYC1  2.18 ± 0.63 | Eef1a1  2.10 ± 0.32 | CYC1  2.52 ± 0.75 | ACTB  3.25 ± 0.72 | LDHA  3.02 ± 0.70 | LDHA  3.02 ± 0.64 | ACTB  3.51 ± 0.69 |
| **5** | HMBS  3.56 ± 0.99 | GUSB  2.64 ± 0.58 | GUSB  2.25 ± 0.49 | CYC1  2.31 ± 0.68 | PPIA  2.52 ± 0.63 | Eef1a1  3.81 ± 0.59 | CYC1  3.11 ± 0.89 | GAPDH  3.89 ± 0.72 | RPL4  4.57 ± 1.11 |
| **6** | HPRT1  3.87 ± 0.90 | GAPDH  2.69 ± 0.50 | HPRT1  2.30 ± 0.52 | HPRT1  2.50 ± 0.59 | GAPDH  3.17 ± 0.55 | GAPDH  3.95 ± 0.68 | PPIA  3.72 ± 0.87 | PPIA  4.83 ± 1.11 | LDHA  4.65 ± 0.94 |
| **7** | ACTB  3.91 ± 0.84 | RPL4  2.79 ± 0.74 | GAPDH  3.40 ± 0.62 | GAPDH  3.18 ± 0.57 | LDHA  3.34 ± 0.74 | HPRT1  4.15 ± 0.99 | GAPDH  3.76 ± 0.67 | HPRT1  5.24 ± 1.15 | GUSB  4.81 ± 1.04 |
| **8** | RPL4  4.25 ± 1.20 | PPIA  3.39 ± 0.80 | RPL4  3.89 ± 1.04 | ACTB  4.08 ± 0.88 | ACTB  3.53 ± 0.75 | RPL4  5.37 ± 1.49 | HPRT1  3.78 ± 0.82 | GUSB  5.77 ± 1.32 | HPRT1  4.81 ± 1.02 |
| **9** | Eef1a1  4.53 ± 0.76 | LDHA  4.05 ± 0.97 | PPIA  4.90 ± 1.15 | RPL4  4.86 ± 1.33 | GUSB  3.77 ± 0.85 | PPIA  5.50 ± 1.44 | RPL4  4.22 ± 1.05 | Eef1a1  5.97 ± 0.91 | PPIA  5.88 ± 1.36 |
| **10** | PPIA  4.53 ± 1.09 | Eef1a1  4.09 ± 0.65 | Eef1a1  5.24 ± 0.81 | PPIA  6.20 ± 1.53 | RPL4  4.31 ± 1.21 | LDHA  7.04 ± 1.56 | Eef1a1  6.41 ± 0.97 | RPL4  9.73 ± 2.50 | Eef1a1  6.11 ± 0.85 |
